# The Australian Genetic Heart Disease Registry: Protocol for a Data Linkage Study

**DOI:** 10.2196/48636

**Published:** 2023-09-20

**Authors:** Alexandra Butters, Bianca Blanch, Anna Kemp-Casey, Judy Do, Laura Yeates, Felicity Leslie, Christopher Semsarian, Lee Nedkoff, Tom Briffa, Jodie Ingles, Joanna Sweeting

**Affiliations:** 1 Clinical Genomics Laboratory Centre for Population Genomics Garvan Institute of Medical Research Darlinghurst Australia; 2 Clinical Genomics Laboratory Centre for Population Genomics Murdoch Children's Research Institute Melbourne Australia; 3 Faculty of Medicine and Health The University of Sydney Sydney Australia; 4 Agnes Ginges Centre for Molecular Cardiology Centenary Institute The University of Sydney Sydney Australia; 5 Clinical and Health Sciences University of South Australia Adelaide Australia; 6 Department of Cardiology Royal Prince Alfred Hospital Sydney Australia; 7 School of Population and Global Health The University of Western Australia Perth Australia; 8 Victor Change Cardiac Research Institute Sydney Australia

**Keywords:** data linkage, genetic heart diseases, health care use, cardiomyopathies, arrhythmia, cardiology, heart, genetics, registry, registries, risk, mortality, national, big data, harmonization, probabilistic matching

## Abstract

**Background:**

Genetic heart diseases such as hypertrophic cardiomyopathy can cause significant morbidity and mortality, ranging from syncope, chest pain, and palpitations to heart failure and sudden cardiac death. These diseases are inherited in an autosomal dominant fashion, meaning family members of affected individuals have a 1 in 2 chance of also inheriting the disease (“at-risk relatives”). The health care use patterns of individuals with a genetic heart disease, including emergency department presentations and hospital admissions, are poorly understood. By linking genetic heart disease registry data to routinely collected health data, we aim to provide a more comprehensive clinical data set to examine the burden of disease on individuals, families, and health care systems.

**Objective:**

The objective of this study is to link the Australian Genetic Heart Disease (AGHD) Registry with routinely collected whole-population health data sets to investigate the health care use of individuals with a genetic heart disease and their at-risk relatives. This linked data set will allow for the investigation of differences in outcomes and health care use due to disease, sex, socioeconomic status, and other factors.

**Methods:**

The AGHD Registry is a nationwide data set that began in 2007 and aims to recruit individuals with a genetic heart disease and their family members. In this study, demographic, clinical, and genetic data (available from 2007 to 2019) for AGHD Registry participants and at-risk relatives residing in New South Wales (NSW), Australia, were linked to routinely collected health data. These data included NSW-based data sets covering hospitalizations (2001-2019), emergency department presentations (2005-2019), and both state-wide and national mortality registries (2007-2019). The linkage was performed by the Centre for Health Record Linkage. Investigations stratifying by diagnosis, age, sex, socioeconomic status, and gene status will be undertaken and reported using descriptive statistics.

**Results:**

NSW AGHD Registry participants were linked to routinely collected health data sets using probabilistic matching (November 2019). Of 1720 AGHD Registry participants, 1384 had linkages with 11,610 hospital records, 7032 emergency department records, and 60 death records. Data assessment and harmonization were performed, and descriptive data analysis is underway.

**Conclusions:**

We intend to provide insights into the health care use patterns of individuals with a genetic heart disease and their at-risk relatives, including frequency of hospital admissions and differences due to factors such as disease, sex, and socioeconomic status. Identifying disparities and potential barriers to care may highlight specific health care needs (eg, between sexes) and factors impacting health care access and use.

**International Registered Report Identifier (IRRID):**

DERR1-10.2196/48636

## Introduction

Genetic heart diseases are a significant cause of cardiac morbidity and mortality in the youth and are characterized by distinct variability in the onset and severity of symptoms [1]. The most prevalent is hypertrophic cardiomyopathy (HCM), which affects up to 1 in 500 people [2]. They are generally inherited as autosomal dominant traits, conferring a 50% risk to first-degree relatives. A diagnosis relies on clinical investigations with a cardiologist and, in some cases, genetic testing, which is ideally conducted in a multidisciplinary clinic [3].

Disease symptoms can be variable ranging from asymptomatic to episodes of unexplained syncope, palpitations, and chest pain to the most serious outcomes of heart failure and sudden cardiac death (SCD). Accordingly, some individuals with a genetic heart disease require life-saving medical intervention via emergency department presentation and hospitalization. Treatment options may include prescription medication, cardioversion, and surgeries such as implantable cardioverter-defibrillator insertion, pacemaker insertion, septal reduction therapies (eg, alcohol septal ablation and septal myectomy), or heart transplant. The implantable cardioverter defibrillator is the only proven therapy to prevent SCD in individuals with a genetic heart disease [4]. The frequency with which these individuals interact with the health care system is unknown, including for noncardiac reasons [5].

Routinely collected health data are data collected for purposes other than research or without specific a priori research questions developed before collection [6]. These data provide both population- and person-based information, meaning we can examine common patterns of health care for the population [7,8]. In jurisdictions with universal health care such as Australia, we can observe an individual’s interaction with hospitals and emergency departments over time and link them to mortality data. Studies that have used routinely collected data to investigate genetic heart diseases, or their consequences such as SCD, focus predominantly on mortality data and may restrict to specific subpopulations such as infants or children [9-11]. There are limitations in correctly identifying individuals with a genetic heart disease in these data collections due to insufficient International Classifications of Diseases (ICD) codes [5], inadequate recognition of these diseases outside of specialized settings, and infrequent interaction with health facilities for many patients who remain largely well and are generally managed via outpatient clinics.

Here we report a protocol for the linkage of Australian Genetic Heart Disease (AGHD) Registry participants [12,13] with state-wide hospitalization, emergency department and mortality registries, and the national mortality registry to establish a rich and comprehensive data resource to examine the health care use of individuals with a genetic heart disease and their families for the first time.

## Methods

### Study Setting, Design, and Population

This is a retrospective cohort study of people in New South Wales (NSW), Australia, enrolled in the AGHD Registry from 2007 to 2019. The AGHD Registry operates nationally and has actively recruited participants since 2007 [12,13]. The goal of the AGHD Registry is to enroll every individual in Australia with a genetic heart disease and their at-risk relatives to understand and improve diagnosis, prevention, and treatment options. Eligibility criteria include a confirmed diagnosis of any inherited cardiomyopathy (HCM, dilated cardiomyopathy, arrhythmogenic cardiomyopathy, and restrictive cardiomyopathy), left ventricular noncompaction, or primary arrhythmia syndromes (long QT syndrome, Brugada syndrome, or catecholaminergic polymorphic ventricular tachycardia; Table 1) or at-risk relatives of those with a confirmed diagnosis.

Participant demographic, clinical, and genetic information (provided by the AGHD Registry data custodian) was linked with whole-population routinely collected health data, specifically hospital admissions (NSW Admitted Patient Data Collection), emergency department presentations (NSW Emergency Department Data Collection), and in the event of death, mortality registry data (NSW Registry of Births, Deaths, and Marriages; Australian Coordinating Registry Cause of Death Unit Record File; Table 2). Although the AGHD Registry is an ongoing registry, linkage to the routinely collected data is not continuous and only occurs on application to the data custodians with ethics approval.

**Table 1:** Classification and description of genetic heart diseases included in the AGHD^a^ Registry.

Genetic heart disease type and specific condition		Definition	Diagnostic tests
**Inherited cardiomyopathies**			
	Hypertrophic cardiomyopathy	Abnormal thickening of the heart muscle on either the left or right ventricle	Electrocardiogram, echocardiogram, cardiac magnetic resonance imaging (MRI), and Holter monitor
	Dilated cardiomyopathy	Enlargement of the left ventricle of the heart affects the ability of the heart to pump blood effectively	Electrocardiogram, echocardiogram, cardiac MRI, and Holter monitor
	Arrhythmogenic cardiomyopathy	A disease of the heart muscle where the normal heart muscle cells on either the left or right ventricle are replaced by fat and scar tissue. This change can lead to heart rhythm abnormalities, or the affected ventricle may become enlarged and not pump blood effectively	Electrocardiogram, echocardiogram, cardiac MRI, and Holter monitor
	Left ventricular noncompaction	Characterized by deep trabeculations (finger-like projections) in the heart muscle wall of predominantly the left ventricle, but may also appear in the right ventricle	Electrocardiogram, echocardiogram, and cardiac MRI
**Primary arrhythmia syndromes**			
	Long QT syndrome	Characterized by abnormal electrical activity in the heart that often presents in children and teenagers	Electrocardiogram, echocardiogram, and exercise or stress test
	Catecholaminergic polymorphic ventricular tachycardia (CPVT)	CPVT is a rare condition often present in children and teenagers. A cardiac event can occur during periods of high emotion or exercise as these situations cause the body to release adrenalin or noradrenalin. These persons have an abnormal response to adrenaline, which causes the heart to beat fast and irregularly	Electrocardiogram, echocardiogram, exercise or stress test, and Holter monitor
	Brugada syndrome	A rhythm disorder that causes the ventricles to beat abnormally fast, affecting the ability of the heart to pump blood effectively	Electrocardiogram, echocardiogram, and drug infusion study

^a^AGHD: Australian Genetic Heart Disease.

**Table 2:** Data sets included in the Australian Genetic Heart Disease (AGHD) Registry data linkage study.

Data set	Purpose	Description	Variables
AGHD Registry	Cohort definition and linked data	National registry collecting clinical and genetic data of patients with genetic heart disease and their families	Demographics Sex Birth date (mm/yyyy) Ethnicity (based on the Australian Standard Classification of Cultural and Ethnic Groups [14]) Vital status (alive or deceased) Postcode Disease status (definite, possible, or at-risk) Smoker status Clinical history Clinical diagnosis Age at diagnosis Reason for diagnosis Genetic status (likely pathogenic, pathogenic variant identified, or not identified) Sudden cardiac events ever Heart failure ever Syncope ever Nonsustained ventricular tachycardia ever Atrial fibrillation ever Left ventricular outflow tract obstruction ever Interventions (implantable cardioverter defibrillator, pacemaker, ablations, myectomy, and heart transplant) Family history Investigations Echocardiography parameters Electrocardiography parameters
NSW^a^ Admitted Patient Data Collection	Linked data	Records all admitted patient services provided by NSW public hospitals, private hospitals, and private day procedure centers	Demographics Sex Birth date (mm/yyyy) Country of birth Residential statistical local area (SLA) at admission Health insurance on admission Episode of care Episode start date Episode end date Condition onset flag Diagnosis codes ICD-AM^b^ (up to 50) Australian Refined Diagnosis-Related Group Hours in intensive care unit Hours on mechanical ventilation Mode of separation Procedure codes ICD-AM (up to 50) Procedure date Service-related group Facility information Hospital type Local health district (LHD) Facility identifier Facility transferred to Facility transferred from
NSW Emergency Department Data Collection	Linked data	Provides information about patient presentations to the emergency departments of public hospitals in NSW	Demographics Sex Birth date (mm/yyyy) Country of birth Residential SLA at admission Health insurance on admission Episode of care Episode start date and time Episode end date and time Diagnosis code ICD-AM (up to 50) Type of visit Mode of separation Referred to on departure Facility information LHD Facility identifier
NSW Registry of Births, Deaths, and Marriages	Linked data	Includes death registrations	Birth date (mm/yyyy) Death date (mm/yyyy) LHD at death
Australian Coordinating Registry Cause of Death Unit Record File	Linked data	Includes death registrations	Death date (mm/yyyy) Death age Place at birth State/territory of usual residence Sex Place of occurrence for external cause of death Underlying cause of death ICD code Contributing cause of death ICD codes (up to 20 comorbidity codes)

^a^NSW: New South Wales.

^b^ICD-AM: International Statistical Classification of Diseases and Related Health Problems, Australian Modification.

### Aims

**Figure 1:**
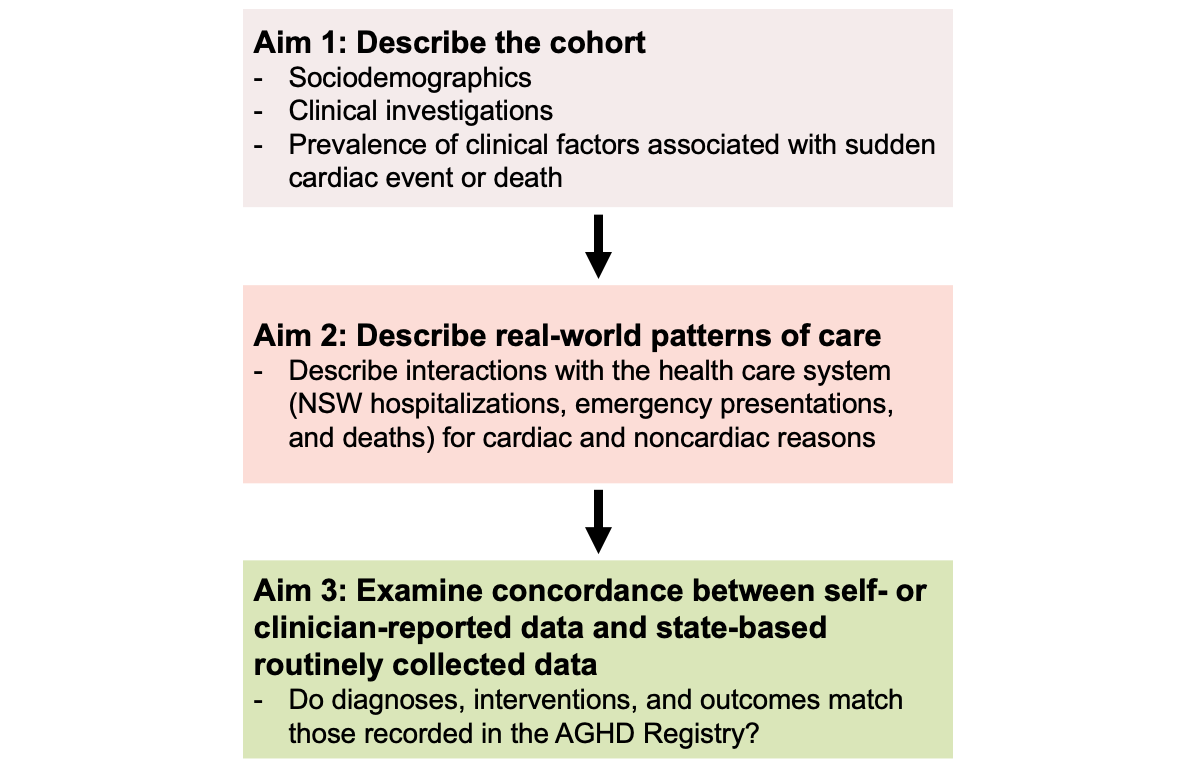
Aims of the Australian Genetic Heart Disease (AGHD) Registry data linkage study. NSW: New South Wales.

**Figure 2:**
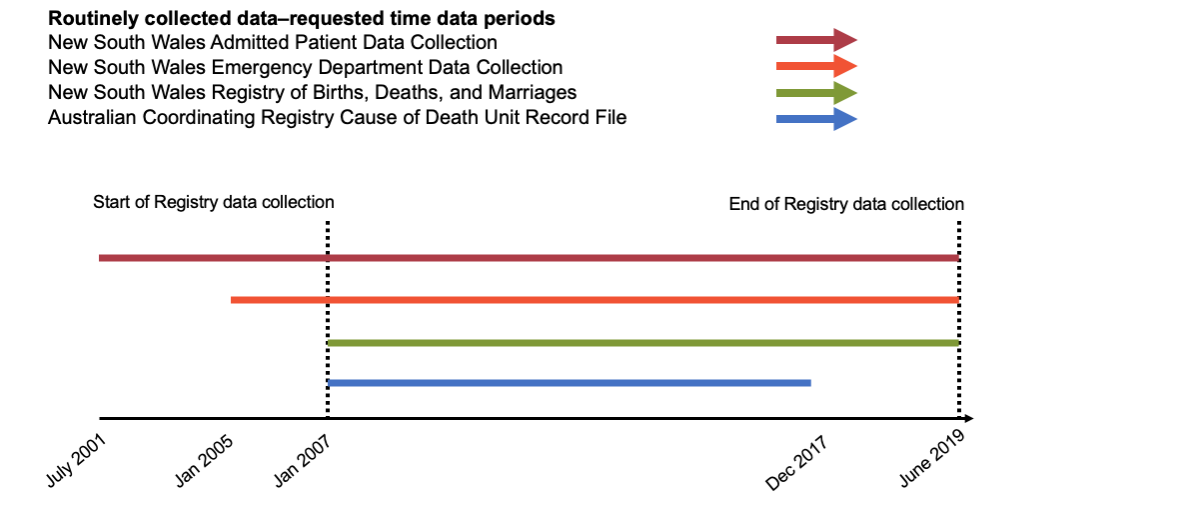
Period of data requested per routinely collected health data set.

Our objective is to characterize AGHD Registry participants using routinely collected whole-population health data sets to investigate the health care use of individuals with a genetic heart disease and their at-risk relatives (Figure 1). We will also examine the concordance between data sources to see whether diagnoses, interventions, and outcomes match those recorded in the AGHD Registry. In our analyses, we will determine if patterns of care vary according to patient demographics such as age, sex, level of residential, and relative socioeconomic disadvantage of patient residence (Figure 2). We will evaluate episodes of care for any medical condition or reason.

### Recruitment and Consent

Participants have been recruited to the AGHD Registry since 2007. Two recruitment methods were used for the data linkage aspects of the study:

1. For participants enrolled in the AGHD Registry between 2007 and April 2017, an opt-out approach was coupled with a waiver of consent. This involved sending a letter to all AGHD Registry participants outlining the study and asking them to contact us if they wish to withdraw their consent for this study. If the participant did not contact us, then the waiver of consent was applied.
2. An amendment to include permission for access to linked data sets was made to the participant information and consent form. Every person enrolled in the AGHD Registry from May 2017 provided written, informed consent for their data to be linked to the administrative data collections mentioned herein.

As part of the AGHD Registry recruitment process, extensive demographic, clinical, and genetic information was collected from the individuals and their cardiologist.

Although participants can withdraw their consent at any time for the AGHD Registry, data already included in the linkage before their withdrawal of consent cannot be removed as all extracted data held by the researchers are deidentified.

### Data Sources and Variables

Table 2 lists and describes the data sets used in this study. The AGHD Registry was used for cohort definition and linking variables with the routinely collected health data. We linked to the following data sets.

#### NSW Admitted Patient Data Collection

The Admitted Patient Data Collection collates information on all admitted patient services provided by NSW public (including psychiatric hospitals and multipurpose services) and private hospitals (including day procedure centers) [15]. Data include patient demographics (ie, sex, birth date, country of birth, and statistical local area of residence at admission), episodes of care (ie, date and time of admission and separation [the completion of treatment for an admitted patient because of death, discharge, or transfer to another facility or care type], referral source, diagnoses, procedures, and service referred upon discharge), and facility information (ie, hospital type, facility, and local health district [LHD]). Up to 50 relevant diagnoses and procedures were included. These are coded according to standardized codes from the International Statistical Classification of Diseases and Related Health Problems, Australian Modification and the Australian Refined Diagnosis-Related Group.

This study included data on admissions between July 1, 2001, and June 30, 2019 (Figure 2). When linked with the AGHD Registry data, this period allowed us to examine patterns of care before and after a genetic heart disease diagnosis. The requested variables will allow us to examine the frequency and type of interactions and care received during hospitalizations.

#### NSW Emergency Department Data Collection

The Emergency Department Data Collection provides information about patient presentations to the emergency departments of public hospitals and contracted private hospitals in NSW [15]. Each record represents a presentation to an emergency department for emergency care and treatment. Data include demographics (same as Admitted Patient Data Collection), episodes of care (eg, date of arrival and separation, diagnoses, and status at separation), and facility information (eg, facility identifier and LHD). Up to 50 relevant diagnoses are included and are coded according to standardized codes from the International Statistical Classification of Diseases and Related Health Problems, Australian Modification.

Data for the Emergency Department Data Collection were available from January 1, 2005, to June 30, 2019 (Figure 2). By combining the hospital data with the AGHD Registry data, we can analyze the emergency department episodes before and after their diagnosis during this period. These data will allow us to explore the frequency and reason for an emergency department presentation for any medical reason.

#### Mortality Data

Two data sets contain mortality information for deaths occurring in NSW: the NSW Registry of Births, Deaths, and Marriages death registrations and the Australian Coordinating Registry Cause of Death Unit Record File [15]. All deaths are certified by a medical practitioner or coroner (if a coronial inquiry is required) and registered by the NSW Registry of Births, Deaths, and Marriages. Details of all registered deaths are forwarded to the Australian Coordinating Registry. Details include the date of death, underlying cause, and up to 20 contributing causes of death. The causes of death are coded using the ICD, Tenth Revision, international coding system rather than the International Statistical Classification of Diseases and Related Health Problems, Tenth Revision, Australian Modification used in Australian hospitals. The Australian Coordinating Registry Cause of Death Unit Record File data are finalized considerably later than the NSW Registry of Births, Deaths, and Marriages death registration data because of the time needed to confirm details on the death certificate.

Data from the NSW Registry of Births, Deaths, and Marriages were available from January 1, 2007, to June 30, 2019, and data from the Australian Coordinating Registry Cause of Death Unit Record File were available from January 1, 2007, to December 31, 2017 (Figure 2). These variables will allow us to determine the reasons and causes of death.

### Data Linkage Methods

Linkage of each AGHD Registry participant’s demographic, clinical, and genetic information with whole-population routinely collected health data was conducted by the Centre for Health Record Linkage (CHeReL). The AGHD Registry data custodian (the individual responsible for the registry data and any related activities) provided CHeReL with 2 separate files. To protect privacy, identifier information, including given names, last names, and current and past residential addresses (street, suburb, postcode, and state), was separated from the content information (registry clinical and genetic information). The 2 files from each data set were stored and handled separately. CHeReL’s data linkage unit applied a combination of matching methods to the identifier data to identify and distinguish between individuals. The identifier data belonging to each individual were then assigned an arbitrary person number, which replaced the name, address, and other identifying details. Using an encrypted version of the arbitrary person number, CHeReL’s data integration unit created a research project-specific person number (PPN). This PPN was used to join the AGHD Registry content data to the NSW state-wide Admitted Patient Data Collection, Emergency Department Data Collection, and the mortality registries (Registry of Births, Deaths, and Marriages and Australian Coordinating Registry Cause of Death Unit Record File). Once linkages were complete, the data sets were released to the research team in a fully deidentified form, ensuring the privacy and confidentiality of all individuals. The AGHD Registry custodian does not have access to the linked data to ensure that the linked data cannot be reidentified. These deidentification measures preclude the withdrawal of any individuals included in the data linkage study after the linkage takes place. Access to the deidentified linked data is only provided to individuals named in the approved ethics protocol. The PPN combines records for the same person across different data sets.

### Statistical Analysis

All data harmonization and analyses will be conducted using the R software (version 4.2.3; R Foundation for Statistical Computing). Harmonization of the data sets will involve systematic identification and removal of any duplicate records, checking for a consistent number of person-level records across the data sets, consistency across variables (eg, admission dates before discharge dates), checking for outliers, and identification and flagging of missing or invalid data. We will analyze and report each data set using descriptive statistics, including mean, median, SD, IQR, and sample size (number). We will also conduct descriptive analysis stratified by factors including age, sex, relative socioeconomic disadvantage, and advantage of patient residence and LHD of facility using the Australian Bureau of Statistics Socio-Economic Indexes for Areas [16] and genetic status (likely pathogenic or pathogenic gene identified vs no gene identified). Additionally, Cox regression models will be developed to identify independent factors associated with adverse outcomes, such as heart failure, atrial fibrillation, stroke, and death.

### Patient and Public Involvement

Patients and the public have not and will not be involved in the study design.

### Ethics Approval

Technical feasibility for the study was granted by the CHeReL, NSW Ministry of Health (ID#2016.38). Ethical approval has been granted by the NSW Population and Health Services Research Ethics Committee (2019/ETH0153) and Sydney Local Health District (Royal Prince Alfred Hospital zone) Human Research Ethics Committee (2020/ETHO00220). The current approval permits data storage at the Garvan Institute of Medical Research. We will disseminate project findings at scientific conferences and in peer-reviewed journals.

## Results

Currently, the research team has received all deidentified data from CHeReL. The record linkage occurred in November 2019. A total of 1384 NSW-based AGHD Registry participants have been linked with 11,610 hospital records, 7032 emergency department records, and 60 death records. Data assessment and cleaning processes have been undertaken, with descriptive data analysis underway. Initial results are expected to be published by early 2024.

## Discussion

This study represents the first in Australia to provide a comprehensive clinical data set on health care use patterns of individuals with a genetic heart disease and their at-risk relatives. It overcomes the limitations of previous studies, which rely on ICD codes to identify patients, by linking data from the AGHD Registry with routinely collected health data in NSW. The study will shed light on health care use among individuals with a genetic heart disease in Australia, examining hospitalizations, emergency department presentations, and mortality rates over approximately 20 years, stratified by clinical diagnosis, age, sex, socioeconomic status, and genetic status. We will investigate the impact of these factors on patterns of care, including interactions with health care providers and on disease severity and outcomes. Overall, we aim to improve the diagnosis, prevention, and treatment options for individuals with a genetic heart disease by providing a better understanding of their health care use and identifying areas for improvement in health care provision. Ultimately, the study’s results will have a significant impact on the care of individuals with a genetic heart disease and their at-risk relatives.

Routinely collected data are commonly used in patterns of care studies. These studies are prolific when investigating cancer populations [17], as they provide the opportunity to describe a patient population and common treatment, as well as examine potential differences such as sex [18] and residential location [19]. In relation to cardiovascular diseases, patterns of care studies have predominantly focused on coronary heart disease or heart failure [18-22]. However, the population of individuals diagnosed with genetic heart diseases differs from those with these conditions, often being younger at diagnosis, with different risk factors and lifestyle constraints such as exclusion from high-level and competitive sports [23]. Therefore, studies such as that described here are needed to examine the unique genetic heart disease population.

By linking the AGHD Registry to routinely collected health data sets, we have overcome a significant limitation of previous studies that relied exclusively on the ICD diagnosis coding system to identify patients with genetic heart diseases [5]. A previous systematic review by our team found that for countries such as Australia, only 3 of the 12 genetic heart diseases included in the search strategy had an explicit ICD diagnosis code [5]. Codes that best match the other 9 genetic heart diseases being investigated were broad descriptive diagnoses (such as other cardiomyopathies used to describe arrhythmogenic cardiomyopathy), which also encompasses other genetic heart diseases. Additionally, the ICD diagnosis coding system fails to explicitly identify rare diseases, with only 500 of the 6000 rare diseases having an ICD diagnosis code [24].

Procedure codes may provide a little more information with specific codes for implantable cardioverter-defibrillator implantation and other interventions and surgeries associated with genetic heart diseases. However, as not all individuals undergo surgical procedures because of their condition, these codes also cannot identify all patients with a genetic heart disease. Because of these coding issues, studies to date have largely focused on those genetic heart diseases where discrimination from linked data sets is possible, that is, HCM and Marfan syndrome.

The clinical course for many patients with a genetic heart disease can be mild, meaning interactions with hospital and emergency departments could be infrequent or not required. However, when hospitalization is necessary, these patients often require urgent and potentially life-saving medical intervention. To date, there is limited research available regarding the health care system interactions of individuals with causative variants, and only one paper has used linked primary care hospital and mortality records in patients with HCM. Our study aims to address these gaps in knowledge and provide answers to these important questions. We will be able to observe the interaction of individuals with less severe clinical course who may never require treatment at a hospital or emergency department for genetic heart disease but present for other reasons.

Because of the absence of studies exploring care patterns associated with genetic heart diseases, it remains unclear whether care varies based on specific factors. Previous studies examining nongenetic cardiac conditions have found that patient factors, such as age, sex, and residential location, impact care patterns [15,18,20,25]. It is vital to examine the impact of socioeconomic factors, as the AGHD Registry contains a significant proportion of patients seen at specialist genetic heart diseases clinics in NSW, which have been shown to overrepresent socioeconomically advantaged individuals [26]. We will be able to investigate if patterns of care or outcomes vary based on the location of the facility and the relative socioeconomic disadvantage of that location. For instance, we will examine whether areas with a relatively high disadvantage have fewer implantable cardioverter defibrillator surgeries than other facilities, as observed in other countries [27].

Among studies using more than one data source, validating the agreement between data types such as self-reporting and routinely collected health data is important. Multiple studies have examined the concordance between these data types for many medical conditions including cardiac diseases [18-21,28-31]. One study found that the agreement between self-report data and health records was substantially lower for persons with heart failure than for other chronic illnesses such as diabetes, hypertension, myocardial infarction, and stroke [28]. Patient factors associated with discordance include sex, age, education level, and socioeconomic status [30,32]. However, no studies have investigated the concordance in individuals with a genetic heart disease between self-reported and routinely collected health data.

There are limitations in coding accuracy, especially for the Emergency Department Data Collection, which could affect the validity of the data collected. The study relies on AGHD Registry participants who are predominantly from a single tertiary center, which has been shown to lack socioeconomic diversity [29]. We will be unable to ascertain the prevalence and incidence of genetic heart diseases accurately as the AGHD Registry does not include all patients in NSW with a genetic heart disease. However, even if our sample is not representative, the relationships between variables are still valid [33].

This study aims to provide valuable insights into the health care use patterns of individuals with a genetic heart disease and their at-risk relatives. By linking data from the AGHD Registry with routinely collected health data in NSW, this study has established a comprehensive clinical data set that can identify reasons for hospitalization and emergency presentation, with the potential to reveal disparities based on disease, sex and sociodemographic status, and opportunities for preventive care. Overall, this study’s findings may have a significant impact on the care of individuals with a genetic heart disease and their at-risk relatives, with the potential for better outcomes and quality of life.

## Abbreviations

AGHD: Australian Genetic Heart Disease
CHeReL: Centre for Health Record Linkage
HCM: hypertrophic cardiomyopathy
ICD: International Classification of Diseases
LHD: local health district
NSW: New South Wales
PPN: project-specific person number
SCD: sudden cardiac death

## Data Availability

The NSW Health data sets generated during and analyzed during this study are not publicly available as they contain sensitive health information and an application must be made to the data custodians to use this data (through the Centre for Health Record Linkage). The Australian Genetic Heart Disease Registry data are available from the Registry custodian (A/Prof Jodie Ingles) on reasonable request.
